# Determination of Tropomyosin in Shrimp and Crab by Liquid Chromatography–Tandem Mass Spectrometry Based on Immunoaffinity Purification

**DOI:** 10.3389/fnut.2022.848294

**Published:** 2022-03-03

**Authors:** Sufang Fan, Junmei Ma, Chunsheng Li, Yanbo Wang, Wen Zeng, Qiang Li, Jinru Zhou, Liming Wang, Yi Wang, Yan Zhang

**Affiliations:** ^1^Hebei Food Safety Key Laboratory, Key Laboratory of Special Food Supervision Technology for State Market Regulation, Hebei Food Inspection and Research Institute, Shijiazhuang, China; ^2^Hebei Key Laboratory of Forensic Medicine, College of Forensic Medicine, Hebei Medical University, Shijiazhuang, China; ^3^Biology Institute of Hebei Academy of Science, Shijiazhuang, China; ^4^School of Food Science and Biotechnology, Zhejiang Gongshang University, Hangzhou, China; ^5^Department of Chemical Engineering, Key Laboratory for Industrial Biocatalysis, Ministry of Education of China, Tsinghua University, Beijing, China

**Keywords:** liquid chromatography-tandem mass spectrometry, tropomyosin, immunoaffinity purification, isotope-label, signature peptide

## Abstract

A UPLC-MS/MS method was developed for the detection of tropomyosin (TM) in shrimp and crab. After simple extraction, the samples were purified by immunoaffinity column and then digested by trypsin. The obtained sample was separated by Easy-nLC 1000-Q Exactive. The obtained spectrums were analyzed by Thermo Proteome Discoverer 1.4 software and then ANIQLVEK with high sensitivity was selected as the quantitative signature peptide. Isotope-labeled internal standard was used in the quantitative analysis. The method showed good linearity in the range of 5–5,000 μg/L with a limit of quantification (LOQ) of 0.1 mg/kg. The average recoveries were 77.22–95.66% with RSDs ≤ 9.97%, and the matrix effects were between 88.53 and 112.60%. This method could be used for rapid screening and quantitative analysis of TM in shrimp and crab. Thus, it could provide technical support for self-testing of TM by food manufacturers and promote further improvement of allergen labeling in China.

## Introduction

Food allergy is an immunoglobulin E-mediated response to food protein, which can lead to anaphylaxis, a life-threatening reaction (1). More and more researchers are paying attention to food allergies for their lethal effects on allergic consumers (2, 3). It is reported that 5% of adults and 8% of young children suffer from food allergies, while more than 90% of food allergies are caused by milk, peanuts, eggs, soybeans, wheat, nuts, fish, and crustaceans (4–9). At the very top of the list of food allergens, shellfish is commonly identified as a cause of food hypersensitivity toward sensitized individuals (10). Allergy to crustacean aquatic products such as shrimp and crab can lead to allergic symptoms such as skin redness and swelling, asthma, and rhinitis, which is accompanied by collapse, shock, and can even be life-threatening, which seriously affects the health and quality of life of allergic people. Shellfish is responsible for approximately 16.1% of all food allergy cases (11, 12). The allergen protein tropomyosin (TM) is a cross reacting allergen among crustaceans and has been shown to be the main allergen in species like shrimp, lobster, crab, and Antarctic krill (13, 14). Studies have shown that the sequence of TM is highly conserved in different shellfish species, that is, there are great similarities in the amino acid sequence of TM in different shrimp and crabs, and there are common epitopes in TM of different crustacean aquatic products (10, 15).

Current analytical methods have been used to determine TM in shellfish products, including enzyme-linked immunosorbent assay (ELISA) (16), quantum-dot-based fluorescent lateral flow immunoassay (LFIA) (17), real-time polymerase reaction (PCR) (18), surface plasmon resonance (SPR) sensor (10), fluorometric sandwich biosensor (12), and aptameric biosensor (19, 20). In recent years, mass spectrometry has been successfully used in the detection of allergens in food due to its high sensitivity and accuracy (21–23), particularly, high-performance liquid chromatography or nanoliquid chromatography–tandem mass spectrometry has been used to determine the intact protein or signature peptides after protein digestion (24, 25).

This experiment was designed to solve the problem of difficult detection of TM in shrimp and crab samples. For the first time, the antibody of TM was obtained and filled to the immunoaffinity column, which was used in the purification of TM. In this study, TM was extracted and purified. The antibody of TM was obtained by immunization, and the immune affinity column of TM was prepared. After simple extraction, the samples were purified by immunoaffinity column and then digested by trypsin. The obtained samples were injected into nanoliquid chromatography–quadrupole/electrostatic field orbitrap high-resolution mass spectrometer for analysis, and the characteristic peptides of TM were obtained after spectrum database retrieval. A liquid chromatography–tandem mass spectrometry method was established with the synthesized characteristic TM peptides as the standard, and the linear relationship, detection limit, method recovery, and other parameters of the established method were verified and applied to the actual sample detection so as to provide technical support for the accurate quantification of TM in shrimp and crab products.

## Materials and Methods

### Chemicals and Reagents

Ammonium bicarbonate (NH_4_HCO_3_), dithiothreitol (DTT), and iodoacetamide (IAA) were supplied by Sigma-Aldrich (St. Louis, MO, USA). TM was prepared and purified in the laboratory. HPLC grade acetonitrile and formic acid were purchased from Merck (Darmstadt, Germany). Tryptase was got from AB Sciex. Distilled water (Watsons, Hong Kong, China) was used in the experiment. Two signature peptides and the internal standard of ANIQLVEK, IVELEEELR, and ANIQL (^13^C_6_,^15^N)VEK were synthesized by Qiangyao Biotechnology Company (Shanghai, China). The purity of all the synthetic peptides was higher than 98%.

HAT, HT, Freund's complete adjuvant, Freund's incomplete adjuvant, PEG4000, and immunoglobulin subtype kit were purchased from Sigma; cell culture plate and DMEM medium were purchased from GIBCO company; HRP-labeled sheep anti-mouse IgG was purchased from Beijing ZhongShan Gold Bridge Biotechnology Limited Company (Beijing, China). Bovine serum albumen (BSA), ovalbumin (OVA), and fetal bovine serum were supplied by Shanghai Shenggong; caprylic acid and ammonium sulfate with analytical grade were obtained from Komil Chemical Reagent Limited Company (Tianjin, China). BALB/c mice aged 6 to 9 weeks were purchased from the Laboratory Animal Center of Hebei Medical University.

### Instruments

Low protein adsorption sample bottles were supplied by Waters. Triple Quad 6500^+^ liquid chromatography–tandem mass spectrometry equipped with MultiQuant 3.0.1 data processing system (AB Sciex, USA) was used for allergen quantification analysis. Orbitrap Fusion equipped with a nanoliquid chromatography system (Easy-nLC 1000) (Thermo Scientific, USA) was adopted for allergen identification.

Carbon dioxide incubator (Sanyo Company, Japan), purification table (Suzhou Purification Equipment limited company, China), inverted microscope (Olympus, Japan), electronic analytical balance (Mettler, Switzerland), liquid nitrogen tank (Chengdu Jinfeng Liquid Nitrogen Container limited company, China), high-speed refrigerated centrifuge (Beckman, Germany), electric thermostatic water bath (Shanghai Jinghong Equipment limited company, China), microplate reader (Bio-Tek, USA), vortex mixer (Shanghai Medical University Instrument Factory, China), and ultramicro nucleic acid protein tester (Thermo, USA) were used in our experiment.

### Preparation of Standard Storage Solution

Proper amount of ANIQLVEK, IVELEEELR, and ANIQL (^13^C_6_,^15^N)VEK were accurately weighed and dissolved in water to 10 ml, and 100 μmol/L mixed standard storage solution was prepared. An appropriate amount of TM was weighed and dissolved in a 10 ml volumetric flask, dissolving it in water to a constant volume, and then 100 μmol/L TM standard storage solution was made.

### Purification of TM

#### Preparation of Acetone Powder

TM was extracted according to the method reported elsewhere (12). The head, tail, and shrimp lines of the white prawns were removed and buffer A (50 mmol/L KCl and 2 mmol/L NaHCO_3_) was added at the ratio of 1:10 (g/ml). The samples were extracted at 4°C for 20 min, centrifuged at 4°C for 10,000 r/min for 20 min, and the precipitates were obtained. The precipitate was resuspended in buffer A of 10 times volume, centrifuged at 4°C for 10,000 r/min for 20 min, and the precipitates were obtained. The above steps were repeated five times. The precipitates were thoroughly washed with precooled acetone until they were colorless, filtered with six layers of gauze, and dried at room temperature. Impurities such as fat and fat-soluble pigment were removed and shrimp acetone powder was obtained.

#### Purification of TM

As described in the literature (12), 1 g acetone powder was weighed and dissolved in buffer B (0.02 mol/L Tris-HCl, 1 mol/L KCl, and 0.1 mmol/L DTT, pH 7.5) with a solid–liquid ratio of 1:5 (g/ml) and extracted for 72 h. The extract was filtered with six layers of gauze and the filtrate was obtained, which was heated for 20 min, centrifuged at 4°C for 10,000 r/min for 20 min after which the supernatant was obtained. Then 30% ammonium sulfate solution was added slowly, placed at 4°C for 1 h, centrifuged at 4°C for 10,000 r/min for 20 min, and the precipitates were got. Then 1 mol/L PBS was used to redissolve it, and 1 mol/L HCl solution was used to adjust the pH to 4.6. Precipitates were got after centrifugation at 4°C for 8,000 r/min for 10 min and PBS was used as the reconstitution solution. The protein content of the complex solution was determined by the BCA method.

### Preparation and Identification of the Monoclonal Antibody

Three female BALB/c mice aged 6–8 weeks were selected for the antibody preparation. TM was diluted with normal saline and injected into the neck and back with an equal volume of Freund's complete adjuvant. The immunization was enhanced every 2 weeks. Freund's incomplete adjuvant of equal volume was added for the second and third immunization. Blood samples were collected after 7–10 days for the third immunization sessions, and indirect ELISA was used to detect the serum antibody titer.

SP2/0 myeloma was fused with spleen cells as described by Yang et al. (26). High-specificity and stable monoclonal cell strains against TM were repeatedly screened out by confirmation detections. The cell strains were cultured to achieve a certain quantity and then injected into atoleine-pretreated BALB/c mice to produce ascites fluids. The ascites fluids were purified according to the octanoic acid–ammonium sulfate method. The TM antibody was freeze-dried and stored at −20°C, and the antibody was thawed and diluted with PBS prior to use. All animal experiments were carried out according to the guidelines of the National Institutes of Health for Care and Use of Laboratory Animals and was approved by the Animal Ethical and Welfare Review Committee of Hebei Food Inspection and Research Institute.

The antibody subtypes were determined using Sigma's immunoglobulin subtype kit. Extract of shrimps, Argentine red shrimps, *Penaeus vannameri*, and bread crab were respectively coated and used to determine the crossreaction of monoclonal antibody by indirect ELISA. The ELISA procedure was as follows: appropriately diluted antigen with a concentration of 5 μg/ml was added to a 96-well ELISA plate (100μl/well) and coated overnight at 4°C, where carbonate buffer was used as the coating solution. After three washings, the wells were blocked with phosphate-buffered saline (PBS) containing 10% gelatin (300 μl/well) at 37°C for 2 h. After washing, 100 μl of the sample extract was added, and the plates were incubated at 37°C for 45 min. Blank control well (PBS) and negative well (negative serum) were set. After washing, 300 μl of 1:10,000 diluted HRP enzyme-labeled goat anti-mouse IgG was added to each well and placed at 37°C for 30 min. After washing, 100 μl of substrate-developing solution was added to each well and reacted at 37°C for 15 min away from light. The A450nm value was measured after the reaction was terminated by the stop buffer.

### Preparation of Immunoaffinity Column

Refer to the instructions of CNBr-activated Sepharose 4B gel, and some modifications were made.

The first step is washing. A total of 2 g CNBr activated Sepharose 4B dry gel was suspended in 20 ml 1 mmol/L HCl to swell, and then the swollen gel was washed with 200 ml 1 mmol/L HCl and fully washed with 200 ml reaction buffer (0.1 mol/L NaHCO_3_ pH 8.1).

The second step is coupling. The pretreated gel was quickly transferred to 10 ml of 2.7 mg/ml TM-antibody coupling buffer (0.1 mol/LNaHCO_3_, 0.5 mol/L NaCl, pH 8.3), stirring overnight at 4°C with the purpose of conjugating TM monoclonal antibody to CNBr-activated Sepharose 4B adequately. The uncoupled antibodies were washed off with a coupling buffer of more than five times the volume of gel and all the eluents were collected. The content of uncoupled proteins in the eluent was determined by ultraviolet spectrophotometer, and the coupling rate was calculated.

The conjugation rate = 1-leached monoclonal antibodies/the added monoclonal antibodies. In the experiment, the conjugation rate between TM antibody and gel was 97.6%.

The third step is washing, where five times of the volume of 0.1 mol/L acetic acid buffer (pH 4.0, containing 0.5 mol/LNaCl) and 0.1 mol/L Tris-HCl (pH 8.0, containing 0.5 mol/LNaCl) were used to alternately wash the gel 4–6 times successively.

The last step is to install the column, where 1 ml column was used to prepare the immunoaffinity column and a sieve was placed into the column. PBS buffer solution (0.01 mol/L pH 7.4) was suspended and the coupling glue was loaded into the column until the glue height was 0.5 ml. PBS was balanced and the bottom of the mouth was sealed and stored in the refrigerator at 4°C.

### Chromatographic and Mass Spectrometry Conditions

#### Easy-nLC 1000-Orbitrap Fusion Conditions

Allergen identification was carried out using Orbitrap Fusion high resolution mass spectrometery system equipped with a nanoliquid chromatography system (Easy-nLC 1000). Precolumn (C_18_, 5 μm, 120A, 100 μm × 4 cm) and analytical column (C_18_, 5 μm, 120A, 75 μm × 15 cm) were supplied by Beijing Lerunfeng Technology Co. LTD. Injection volume was 2 μl, the flow rate of the sample pickup was 20 μl/min, and the volume of sample loading was 20 μl. Mobile phase A was water with 0.1% formic acid and mobile phase B was acetonitrile. Precolumn equilibration was conducted by 8 μl mobile phase A and analytical column equilibration was carried out by 6 μl mobile phase A. Gradient elution was adopted, the elution program was set up with a linear gradient from 3% B to 7% B in 3 min, gradient to 22% B in 38 min, gradient to 35% in 48 min, ramped to 90% B in 50 min, then held at 90% B for 10 min, and the flow rate was 300 nl/min. It takes 70 min to complete one analysis.

All data were acquired in full-MS and data-dependent scan (ddMS^2^) mode under the electrospray positive ion mode. Full MS conditions were set as follows: resolution, 1,20,000; AGC target was set as 2e5; and scan range was 350–1,500 m/z. dd-MS^2^ conditions were set as follows: isolation mode was set as quadrupole; activation type was set as HCD; resolution, 15,000; and AGC target was set as 5.0e4. Isolate window was 1.6 m/z, fixed first mass was 100.0 m/z, and HCD collision energy was set as 30%.

#### Triple Quad 6500^+^ Conditions

A Triple Quad 6500^+^ liquid chromatography–tandem mass spectrometry was used for allergen quantification. A LC-30AD UPLC system equipped with binary solvent manager, sample manager, and column manager was adopted for the tryptic peptides separation (Shimadzu, Japan). The column was XBridge BEH C_18_ (2.5 μm, 2.1 mm × 100 mm, Waters). Then 0.1% formic acid (mobile A) and acetonitrile (mobile B) were used as mobile phase with a flow rate of 0.3 ml/min, the column temperature was set at 40°C, and the injection volume was 1 μl. Mobile phase B was maintained at 2% in the initial 1.0 min, linear gradient from 2 to 65% in 7.0 min, and then held at 65% for 2.0 min, returned back to 2% B in 0.01 min, and equilibrated at 2% B for 2.0 min.

The ESI source was used in data acquisition of the Triple Quad 6500^+^ MS and multiple reaction monitoring (MRM) was used as scan mode. The capillary voltage was set as 5.5 kV, the pressure of atomizer (GS1) was set as 50 psi, the pressure of auxiliary gas (GS2) was set as 55 psi, the pressure of curtain gas was 30 psi, and the temperature of ion source (TEM) was 500°C.

### Data Analysis

Raw data obtained from Orbitrap Fusion was analyzed by the software Thermo Proteome Discoverer 1.4, and the relative parameters were set as follows: MS1 precursor was adopted in precursor selection; minimum precursor mass was set as 350 Da; maximum precursor mass was set as 5,000 Da; the minimum peak count was 1; protein database, shrimp database, and crab database were downloaded from Uniprot (http://www.uniprot.org); trypsin was used in enzymatic hydrolysis; maximum missed cleavage site was set as 2; minimum peptide length was 6; maximum peptide length was set as 144; the precursor mass tolerance was 10 ppm; fragment mass tolerance was 0.02 Da; oxidation (+15.995 Da) was selected in dynamic modification; and carbamidomethyl (+57.021 Da) was chosen in static modification.

### Sample Extraction and Purification

Solid samples were finely ground before extraction as reported before (27). Then 0.1 g of the homogenized sample was weighed, 10 ml distilled water was added, the sample was shaken for 1 h, and centrifuged for 10 min with 9,500 g. Then 3 ml extract was taken for subsequent purification treatment.

The immunoaffinity column was taken out and the plug was removed to allow the liquid to flow out by gravity. A total of 10 ml phosphate buffer was used to balance the immunoaffinity column when the fluid in the column was no longer dripping. A total of 3 ml extract was sampled after the phosphate buffer was close to the sieve plate. Then 10 ml phosphate buffer (PH 7.4) was added to wash the impurities in the affinity column after the level of the sample liquid entered the affinity column. After the level of the sample liquid entered the affinity column, 10 ml phosphate buffer (PH 7.4) was added to wash the impurities in the affinity column.

After leaching, phosphate buffer was removed clearly with washing ears ball or cylinder. A total of 2.7 ml elution buffer (0.1 M of glycine buffer, pH 2.5) was added as the eluent solution by gravity flow rate drop out and the flow rate should be less than one drop each second. After all the liquid falls into the sieve plate, the eluent in the column was brought out by the ear ball or needle. A total 300 μl neutralization buffer (1 M Tris, pH 9.0) was added to the eluent, quickly mixed with the liquid collected in the test tube for subsequent enzymatic hydrolysis treatment.

### Enzymatic Digestion

Enzymatic digestion was conducted according to literature report (21). An aliquot of 500 μl sample solution was spiked with 25 μl of 25 nM stable isotope-labeled internal standard solution and then mixed with 10 μl of 500 mM DTT solution in a 60°C water bath for 30 min. An alkylation was performed with the addition of 30 μl 500 mM IAA solution at room temperature for 30 min in the dark. Thereafter, 100 μl 500 mM NH_4_HCO_3_ solution and 40 μl of 250 μg/ml trypsin were added and incubated overnight at 37°C in a water bath vibrator. Then, 20 μl 0.1% formic acid was added to terminate the digestion and 275 μl pure water was added to fill the volume to 1 ml. The sample was centrifuged at 14,000 g at room temperature for 20 min and the supernatant solution was collected for determination.

### Statistical Analysis

The statistical significance was analyzed through one-way analysis of variance by IBM SPSS Statistics 26. All experiments were performed at least three times.

## Results and Discussion

### Antibody Production and Characterization

The characteristics of the TM antibody were evaluated by indirect competition ELISA (28, 29). The serum titers of immunized mice were more than 1:4,00,000. Seven cell lines of anti-shrimp TM monoclonal antibody were screened, which were named as 1H1, 2C1, 2H11, 2F12, 3B2, 3H11, and 4B9, respectively. The protein concentration was measured by ultrafine nucleic acid protein analyzer and the titer was determined by indirect ELISA. The results showed that 2H11 had the highest titer, which was 1:1 million, the titer of 1H11, 3H11, and 4B9 was more than 1:250 000, and the titer of 2C1, 2F12, and 3B2 was more than 1:30 000. 2H11 was selected in the preparation of the subsequent immunoaffinity column.

The extracts of base shrimp, Argentine red shrimp, *P. vannamei*, and bread crab (labeled TMJ, TMA, TMN, TMM, respectively) and TM, 2H11 was diluted at 1:200, 1:50000, and 1:200000 times, respectively, and indirect ELISA was used to detect the crossreaction rate. The crossreaction rate was calculated by A450nm (sample)/A450nm (TM) × dilution ratio. The results were shown in Table 1. When the dilution factor was 200, the crossreaction rates were higher than 86.5%; when the dilution factor was 50,000, the crossreaction rate of TMJ and TMA was 77.5%, 39.2%, respectively, and the crossreaction rate of TMN and TMM was lower than 20%. When the dilution factor was 2,00,000, the crossreaction rate of TMJ was 53.2%, the crossreaction rate of TMA was 25.3%, and the crossreaction rate of TMN and TMM was lower than 10%. The results showed that when the dilution factor was low, the crossreaction rate was serious, which indicated the high homology of TM in different shrimp and crab. Significant differences existed in the crossreaction rates among different species at different dilution ratios, and the *p*-values were lower than 0.001.

**Table 1:** The results of crossreaction of antibody 1H11 with four analogs (*n* = 3).

**Cross reactant**	**Dilution factor was 200**		**Dilution factor was 50,000**		**Dilution factor was 200,000**	
	**A_**450nm**_**	**Cross reaction rate (%)**	**A_**450nm**_**	**Cross reaction rate (%)**	**A_**450nm**_**	**Cross reaction rate (%)**
TM	2.706 ± 0.0096	100	2.607 ± 0.0076	100	1.560 ± 0.0038	100
TMJ	2.581 ± 0.0020	95.4 ± 0.223	2.020 ± 0.0062	77.5 ± 0.255	0.830 ± 0.0025	53.2 ± 0.083
TMA	2.454 ± 0.0098	90.7 ± 0.178	1.023 ± 0.010	39.2 ± 0.287	0.395 ± 0.0076	25.3 ± 0.425
TMN	2.488 ± 0.0080	91.9 ± 0.501	0.460 ± 0.0022	17.6 ± 0.0607	0.145 ± 0.0060	9.3 ± 0.364
TMM	2.342 ± 0.0040	86.5 ± 0.355	0.406 ± 0.0051	15.6 ± 0.234	0.123 ± 0.0050	7.9 ± 0.307

*The results indicate with “mean ± SD”*.

### Capacity Test of Immunoaffinity Column

To test the column capacity of self-made immunoaffinity column, we tested the recovery rate of different sample loading. As shown in Table 2, when the loading quantity of TM was in the range of 40–80 μg, the recovery rate was above 95%, and when the loading quantity reached 100 μg, the recovery rate was 79.1%. This is because the immunoaffinity column purification is based on the specific binding of antigen and antibody. Therefore, when the amount of antibody filled in the immunoaffinity column is fixed, the amount of antigen that can be adsorbed has an extreme limit. When all the specific binding sites of antibody are occupied, the specific binding of more antigens cannot be carried out. To ensure the purification effect, it is recommended that the sample quantity is not higher than 80 μg.

**Table 2:** The results of column capacity test with different sample loading quantity.

Sample loading quantity (μg)	40	50	60	80	100
Recovery (%)	95.8	96.3	96.5	95.2	79.1

### Selection and Synthesis of Signature Peptide for Allergen Protein

The selection of signature peptides is very important in the LC-MS/MS method development, which could influence the specificity and sensitivity of the method for the different ionization response of different peptides (24). Trypsin was used in enzymatic hydrolysis, where the cleavage site of trypsin was specifically at the C-terminal of lysine and arginine (30). When the length of peptide is lower than five amino acids, the analytical specificity is poor; when the peptides are too long, it is difficult and expensive to synthesize and the response of mass spectrometry is also unfavorable, so the peptide length is usually seven and 16 amino acids. To prevent possible chemical modifications, some susceptible amino acids, such as cysteine and methionine, should be avoided in the signature peptides selection. The selected peptide should be reproducibly observed and detectable in different states of samples, including the digested sample (31, 32).

Nanoliquid chromatography system tandem Orbitrap Fusion was used to analyze the peptide fragments after enzymolysis of TM. Raw data was analyzed by Thermo Proteome Discoverer 1.4. Twenty-eight peptides of TM were identified, and ANIQLVEK (AK-8), IVELEEELR (IR-9) were selected as signature peptides according to the principles selection.

Internal peptide (IP) was designed in our method. Based on the enzymatic digestion technique, homologous peptide was employed as the IP for measuring shrimp and crab allergen proteins. The isotopically labeled IP ANIQL (^13^C_6_, ^15^N)VEK was designed and synthesized, in which all the carbon and nitrogen atoms in leucine (L) residues were labeled with ^13^C and ^15^N.

### Optimization of MRM Conditions

**Figure 1:**
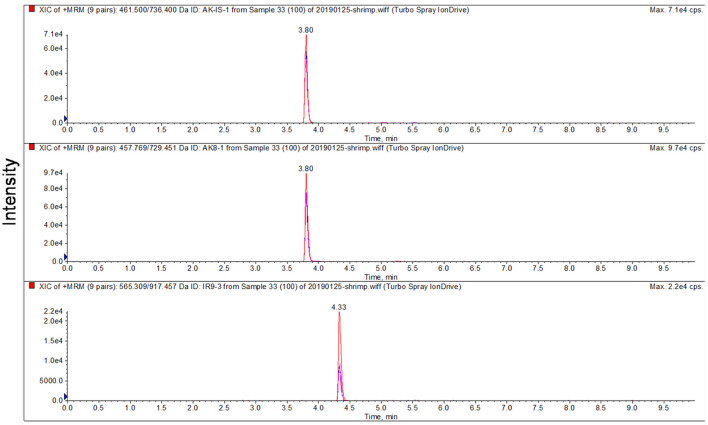
Chromatographic-mass spectrograms of signature peptides and internal standard of tropomyosin.

To optimize the parameters of mass spectrometry, standard solutions of synthetic peptides were directly injected into mass spectrometry by the syringe pump. Full scan mode was used to find the precursor ion of peptide fragment, the declustering potential was also optimized, the product ions were confirmed in product scan mode, and the collision energy was optimized. Three product ions were selected for each precursor ion and precursor-to-product ion transitions were detected by multiple reaction monitoring (MRM) mode. AK-8 was selected as the quantitative peptide for TM. All parameters of MS are shown in Table 3. The chromatographic-mass spectrograms of signature peptides and internal standard of TM are shown in Figure 1.

**Table 3:** Mass spectrometry parameters of signature peptides and the isotope-labeled internal standard of tropomyosin.

**Allergen**	**Peptide**	**Q_**1**_ (m/z)**	**Q_**3**_ (m/z)**	**Fragmenter (V)**	**CE (eV)**
Tropomyosin	ANIQLVEK^&^	457.769	729.451^*^	80	21.4
			616.366		21.4
			488.308		21.4
	IVELEEELR	565.309	917.457^*^	80	26.7
			788.415		26.7
			675.311		26.7
	ANIQL (^13^C_6_,^15^N)VEK	461.500	736.400^*^	50	18.3
			623.300		19.0
			495.300		21.8

& *Marked for quantitative peptide*.

* *Marked for quantitative ions*.

### Method Validation

#### Specificity of the Method

The specificity of the method means that the method should not be interfered with nontarget subjects. Both the peptide standards and the tryptic samples spiked with the internal standard were detected to investigate the specificity of the method. There were sharp and symmetric peaks in the synthetic peptide standards and the selected signature peptide from tryptic samples, whereas there were no peaks in samples without tryptic digestion. There were no interferences from the matrix components on the retention time of the peptide standards, which indicated the perfect specificity of the method (32).

#### Matrix Effect

Matrix effect must be considered in mass spectrometry, which means the change in the analytical signal is caused by anything in the sample matrix (33). Signal suppression or enhancement of the analyte due to the coelution of matrix components could influence the accuracy of the method (34–36). Matrix effect could be caused by compounds brought from complex matrices of analytical samples, solvents, reagents, and materials used in sample preparation or solvents, buffers, and additives contained in the mobile phase. Postextraction addition, post-column infusion, and comparison of slopes of calibration curves are the main approaches to evaluate the matrix effect (37).

In this experiment, matrix effects were expressed as the ratio between the calibration curve slopes of matrix-matched and solvent-based standards. If the percentage of these slopes is larger than 100%, signal enhancement would occur, and when the percentage is lower than 100%, signal suppression may exist (38). In our test, the matrix effects of potato chips and sea bass substrate without shrimp were tested, and the results showed that the matrix effects of potato chips and sea bass were 92.8 and 98.6%, respectively. Therefore, in the follow-up experiment, the matrix-matched standard curve was used in quantitative analysis to compensate for the matrix effect. In general, the internal standard method was not significantly influenced by the matrix effect.

#### Linear Range, Limit of Detection, and Limit of Quantification

In order to investigate the linearity, limits of detection (LODs) and limits of quantification (LOQs) of the method, potato chips and sea bass without shrimp were subject to method validation. The standard curves were fitted between the analyte/IP peptide area ratio (y) versus analyte/IS concentration ratio (x). The concentrations of synthetic peptides ranged from 0.5 to 400 nmol/L of TM in different matrices, with correlation coefficients (*R*^2^) higher than 0.999 in all cases. Blank substrate of potato chips and sea bass without shrimp were used to test the LODs and LOQs using the spiked samples. The spike levels of target peptides with signal-to-noise ratio of three and 10 are defined as the LOD and LOQ of the method. The LOD and LOQ were expressed as TM contents, which were calculated based on the equimolar relationship between the protein and signature peptide. The LOD of TM in potato chips and sea bass substrate was 7.16 and 3.58 μg/g, respectively. The LOQ of TM in potato chips and sea bass substrate was 14.3 and 7.16 μg/g, respectively. Data of linear range, regression equation, LOD, and LOQ are presented in Table 4.

**Table 4:** Linearity, LOD, and LOD of the method.

**Matrix**	**Linear range (nmol/L)**	**Regression equation**	***R* ^2^**	**Limit of detection (μg/g)**	**Limit of quantification (μg/g)**
Potato chip	0.5~400	Y = 0.33958X + 0.01748	0.99950	7.16	14.3
Sea bass	0.5~400	Y = 0.36087X – 0.02017	0.99919	3.58	7.16

#### Method Recovery and Precision

Method accuracy was confirmed by spike samples, while the precision of the method was studied by carrying out five parallels of each spiking level. Precision of the method was expressed by relative standard deviation (RSD). The recoveries of TM for different matrices were calculated based on the samples spiked at three levels on LOQ, 3 LOQ, and 10 LOQ, from which the recovery of TM was determined in a range of 84.3–92.8% and RSDs were in the range of 1.32–5.24%. The data of recovery and precision were given in Table 5.

**Table 5:** Recovery and precision of the method (*n* = 5).

**Matrix**	**Spike level (μg/g)**	**Recovery (%)**	**RSD (%)**
Potato chip	14.3	87.5	4.66
	42.9	92.7	1.32
	143	92.8	1.48
Sea bass	7.16	84.3	5.24
	21.5	89.9	2.32
	71.6	92.2	1.65

### Sample Analysis

A total of 13 samples purchased from local supermarkets were used to determine the applicability of the method, including prawn, shrimp sticks, shrimp balls, crab stick, fish balls, etc. As shown in Table 6, TM was detected in *Penaeus vannamei* and China shrimp samples, with a concentration of 3,291 and 3,730 μg/g. TM was not detected in other samples. The results showed that the developed method could be used for the determination of TM in different kinds of food samples.

**Table 6:** Results of real samples.

**Sample**	**TM (μg/g)**
Penaeus vannamei	3,291
China shrimp	3,730
Fresh shrimp slices	ND
Fresh shrimp strips	ND
Shrimp balls	ND
Lobster steak	ND
Lobster stick	ND
Crab king stick	ND
Crab chops	ND
Fish ball	ND
Cuttle ball	ND
Dragon prawn ball	ND

### The Advantages of the Method

To compare relevant papers in the field, immunoaffinity purification was used in the procedure of sample pretreatment for first time. TM extraction, purification, antibody preparation, and the preparation of immunoaffinity column were finished by members of the research group. Signature peptides and isotope-labeled internal standard were used in the quantitative analysis. The method has the advantages of simple pretreatment, less interference, high specificity, and perfect accuracy and precision.

## Conclusion

In this experiment, TM was extracted and purified, antibody of TM was made, and immunoaffinity column was filled. After simple extraction, the samples were purified by immunoaffinity column and then digested by trypsin. A new liquid chromatography–tandem mass spectrometry method at the peptide level was developed to determine TM, NIQLVEK (AK-8) was confirmed as the quantitative peptide and synthesized for the further process, and isotope-labeled internal standard was used in the quantitative analysis. The specificity, linearity, sensitivity, matrix effect, accuracy, and precision of the method were investigated, and the developed method has been successfully used for the detection of TM in various food samples.

## Data Availability Statement

The raw data supporting the conclusions of this article will be made available by the authors, without undue reservation.

## Ethics Statement

The animal study was reviewed and approved by Animal Ethical and Welfare Review Committee of Hebei Food Inspection and Research Institute.

## Author Contributions

YZ and YW conceived and designed the experiments. SF, JM, QL, CL, and JZ performed the experiments. YW analyzed the data. WZ and LW wrote the original draft. All authors have read and approved the manuscript.

## Funding

This work was supported by Hebei Province High-Level Talent Funding Program (Project No. A201901008), Science and Technology Project of State Administration for Market Regulation (Project No. 2021MK023), and Research Project of Hebei Administration for Market Regulation (Project No. 2020ZD12).

## Conflict of Interest

The authors declare that the research was conducted in the absence of any commercial or financial relationships that could be construed as a potential conflict of interest.

## Publisher's Note

All claims expressed in this article are solely those of the authors and do not necessarily represent those of their affiliated organizations, or those of the publisher, the editors and the reviewers. Any product that may be evaluated in this article, or claim that may be made by its manufacturer, is not guaranteed or endorsed by the publisher.
